# From tradition to innovation: a comparison of the traditional 4-step approach versus a blended learning modification for technical skills teaching

**DOI:** 10.1186/s13049-023-01127-4

**Published:** 2023-11-14

**Authors:** Elonka Bergmans, Alistair Billington, Karl-Christian Thies

**Affiliations:** 1 Klinik für Anästhesiologie, Intensiv-, Notfallmedizin, Transfusionsmedizin und Schmerztherapie, Ev. Klinikum Bethel, Universitätsklinikum OWL der Universität Bielefeld, Campus Bielefeld, Bethel, Burgstieg 13, 33617 Bielefeld, Germany; 2 European Trauma Course Organisation, PO Box 452, Market Drayton, TF9 9FB UK; 3 Centre for Medical Education, University of Dundee, Nethergate, Dundee, Scotland UK

**Keywords:** 4 step technique, Blended learning, Flipped classroom, Pelvic binder

## Abstract

**Background:**

This experimental study was performed to evaluate the role of blended learning for technical skill teaching on the European Trauma Course (ETC). While online modules are extensively used for theoretical teaching, their role in skills training remains less well explored. The ETC currently relies on the established 4-step technique for teaching technical skills. However, the required large cohort of skilled instructors and the time intensity prove increasingly challenging in a current climate of staff shortages and funding constraints. This study assesses if blended learning, combining pre-course online elements with face-to-face training matches the effectiveness of the traditional 4-step approach whilst being more time-efficient.

**Methods:**

In a randomised, multi-centre trial, the conventional face-to-face 4-step technique for teaching a skill of medium complexity, the application of a pelvic binder, was compared with an innovative blended approach. It was hypothesised that the blended approach was non-inferior for skill performance measured after the teaching session and after two days (skill retention) with the time needed for teaching and student/teacher satisfaction as secondary outcomes.

**Results:**

Ninety participants, divided into 44 traditional and 46 blended method students, were analysed. Independent-samples t-test showed no significant difference in performance scores and non-inferiority of the blended approach with a half of one standard deviation margin. A statistically significant difference in mean retention scores favored the blended approach. A Mann–Whitney U Test revealed no significant difference in candidate satisfaction levels but a statistically significant difference in instructors' satisfaction levels in favour of the blended approach. Analysis with Welch' t-test demonstrated that the face-to-face teaching time needed for the blended approach was significantly shorter (by 6 min).

**Conclusions:**

The integration of a blended approach with the 4-step technique for teaching pelvic binder application in the ETC streamlined teaching without compromising skill acquisition quality. This innovative approach addresses traditional limitations and shows promise in adapting medical education to modern learning and teaching demands. We suggest that blended learning could also be applied for other skills taught on life support courses.

*Trial registration:* University of Dundee (Schools of Medicine and Life sciences Research Ethics Committee, REC number 22/59, 28th June 2022).

**Supplementary Information:**

The online version contains supplementary material available at 10.1186/s13049-023-01127-4.

## Background

Drawing from the lessons of the Covid pandemic, the International Liaison Committee on Resuscitation (ILCOR) has advised that all life support courses, including the European Trauma Course (ETC), embrace blended learning methodologies to enhance the resilience of teaching and training [1]. However, changing the approach to teaching life-saving trauma skills in an accredited life support course like the ETC requires a scrutinised process. The recent ILCOR systematic review showed that a blended approach for basic and advanced life support skills was comparable to traditional teaching methods with regards to performance [1] but the level of evidence was low. Only one study, using knowledge level rather than skill performance as outcome parameter, reported on a blended approach to trauma life support teaching [2].

An experimental study was devised to address the current evidence gap by evaluating the role of blended learning for technical skill teaching in the ETC. The ETC currently uses the 4-step technique [3] for teaching technical skills, which has been reported as superior to other teaching techniques with respect to skill acquisition and retention [4]. The 4-step approach consists of:

- Step 1: Real time teacher demonstration of the skill
- Step 2: Explanation of the different skill steps
- Step 3: Learner talking the teacher or peer learner through the steps whilst the teacher/peer learner performs them
- Step 4: Learner performs the skill steps

Unfortunately, when taught in small groups [5] this technique may not suit candidates' needs with some feeling overwhelmed with information whilst others are bored [6]. Furthermore, the technique requires a large cohort of well-trained instructors and is time-consuming, especially for more complex skills [7]. Replacing part of the face-to-face teaching with an online module while maintaining the 4-step structure, would provide learners with a more flexible and self-paced mode of learning. Face-to-face time can then be used for skill practice and feedback to help mastering the skill. This blended learning model, in which e-learning is completed independently before class in order to apply the learned concepts during class, is called the flipped classroom [8]. The flipped classroom concept significantly improved student learning, assessed by knowledge tests or Objective Structured Clinical Examination, compared to traditional teaching methods [9]. Furthermore, standardised online teaching resources can bolster teaching consistency, ensuring quality control of teaching content. Such materials are not only easier to update but could also potentially reduce the duration of face-to-face instruction. The presented study compared the ETC ‘standard’ method of skill teaching with a new blended approach, based on the flipped classroom model described above. Placement of a pelvic binder was picked as the technical skill to be investigated. Although the steps of this skill are relatively straightforward, experience showed that the correct completion of all steps was a recurrent issue in the summative course assessment of the ETC. The skill was judged to be not too complex as the nine skill steps lie within the recommended maximum number of steps to be taught in one session [10].

The aim of the study was to determine if a blended approach can be equally effective and more time-efficient than the traditional 4-step approach.

## Study methodology

Based on a post-positivist paradigm, this quantitative, experimental study compared two teaching techniques for their effectiveness. The hypotheses state that the blended approach will be non-inferior to the traditional approach in terms of skill performance, skill retention, student and teacher satisfaction and additionally will need less face-to-face teaching time compared to the traditional technique.

The sample size was calculated based on previous skill performance score distributions of checklist ratings [6, 11] combined with the insights of five ETC instructors regarding current pelvic binder application criteria. The study was designed to identify a 4-point score difference (25% of total score) between two groups, with 80% power, a 0.05 alpha level, and a common standard deviation of 6. The resulting sample size required was 35 for each group.

The study population was made up of ETC candidates. Centres in England (Birmingham, Manchester, Stafford) and Belgium (Antwerp, Ghent) were selected for participation based on course availability, willingness to participate and researchers' travel duration. All candidates were contacted via email one month before their course. Prior exposure to any form of pelvic binder instructions was not counted as an exclusion criterion as the majority of ETC candidates work in trauma care and are expected to have had some exposure to pelvic binder application.

The standard ETC Master Data File (MDF) on Microsoft Excel 2016 was used to randomly allocate participants to one of the two study groups. Participant information and consent forms kept details about the study hypotheses and teaching techniques confidential, blinding participants to these aspects. Instructors were manually assigned to the teaching sessions by a blinded course director. Double blinding was not possible due to the need for instructors to be informed about both teaching techniques to safeguard teaching consistency during the course.

### Design

#### Setting

The study occurred over five courses, each lasting 2.5 days. Pelvic binder teaching sessions took place on the first day, with 2 to 4 instructors teaching groups of 4 candidates. Sessions for control and research groups were conducted simultaneously in different rooms, and instructors taught the same session three times.

#### Procedure for the control – and research group

**Fig. 1:**
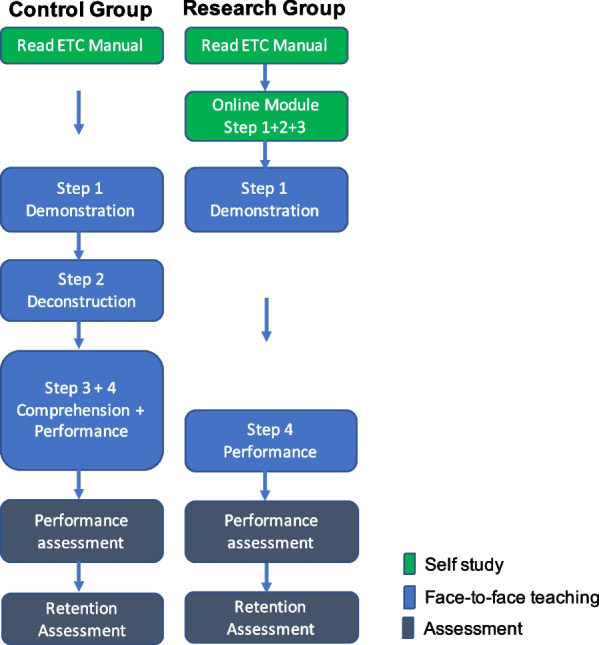
Study design. Two groups received skills training for pelvic binder application; a face-to-face 4-step approach for the control group and a blended modification for the research group. Both groups were assessed for their performance immediately after the teaching session and 2 days later (retention). Further outcome measurements were the time needed for teaching and learner/teacher satisfaction

Figure 1 illustrates the distinct teaching approaches for the control and research group.

#### Self-study for both groups

Candidates were expected to read the ETC course manual's pelvic binder instructions, before the course.

#### Online module for research group

The research group received an additional online module two weeks before the course, including theoretical content, videos showing step 1 and 2, online activities for step 3, and self-assessment questions.

All instructors received the online module, with the instructors for the control group instructed to teach the skill as shown in the online module to standardise teaching.

#### Face-to-face teaching

Step 1 for both groups was a real-time demonstration of pelvic binder application during a simulated trauma scenario, aligning with the concept of contextual learning [12].

Steps 2–4 for the control group involved a detailed explanation, followed by candidates guiding each other through the application with corrective feedback from the instructor and further time to practice.

The research group, having completed steps 2 and 3 online, immediately performed step 4 with corrective feedback from the instructor and practice time.

Instructors measured the time required to teach pelvic binder application. The control group's time covered steps 2 to 4, while the research group's time only included step 4. Step 4 was deemed complete when candidates felt competent to perform the skill. Although the control group had more steps, the effectiveness of the online module in preparing the research group was uncertain, possibly leading to a much longer step 4 for the research group due to more corrections and questions.

#### Assessment

After step 4, instructors assessed candidates' skill performance using a standardised checklist, which was based on the regular ETC criteria and the European Trauma Course Manual [13] and modified after consultation with 10 experienced ETC instructors for content and face validity (Additional file 1). Like prior studies [6, 14], a trinary scoring scale was used.

#### Evaluation

After the teaching session, candidates were asked to rate their satisfaction with the teaching technique on a 5-point Likert scale, covering the aspects of explanation, feedback, practice time, and assessment objectivity (Additional file 2). For the research group, this included both online and face-to-face teaching. Additional questions covered prior training exposure and time spent on the online module.

Instructors also rated their satisfaction on a 5-point scale, focusing on time for feedback and practice, objectivity of assessment, and comparing blended learning to the standard technique if in the research group (Additional file 3).

#### Retention

All candidates completed a second performance assessment (retention assessment) on day three in a separate room with an assessor, a research assistant and a manikin.

## Data collection and analysis

Data collection was subjected to the European General Data Protection Regulation (2016). Data were analysed using SPSS Statistics version 29 (IBM). Descriptive statistics were given in terms of frequencies, means, medians, standard deviation, and range. Independent samples t-tests were used for performance and retention comparisons between groups, with non-inferiority calculations defining a margin of half a standard deviation [15]. Satisfaction questionnaires were analysed using the Mann-Whitney U Test, with the effect size reported as R-value, representing r = 0.1 as a small, r = 0.3 as a medium and r = 0.5 as a large effect. For time measurement comparisons Welch's t-test was used as Levene's test indicated that the assumption of equal variances was not met for this variable. Cohen's d was calculated for the effect size.

## Results

### Participants

A total of 108 candidates were invited to take part in the study and all agreed to participate by signing informed consent. Four participants were lost to attrition because they did not attend the course. Two participants from the control group were excluded because they had viewed the online module which a fellow participant of the research group had shared with them. Twelve participants were excluded because the instructor group deviated from the research protocol by including a different pelvic binder model into the teaching session. The remaining 90 study participants, were divided between traditional teaching (n = 44) and a blended approach (n = 46).

Completion time of the online module varied, with 32 candidates taking 0–15 min and 10 candidates taking 15–30 min; data for 2 were missing. Previous exposure to training on the application of a pelvic binder was nearly equal between groups, with 25 in the control group and 28 in the research group, constituting 61% of each group. Data for 3 candidates in the control group were missing.

### Performance score as outcome parameter

Both the control and research groups scored highly on the performance assessment, with mean scores of 17.07 (SD = 1.58) and 17.43 (SD = 0.98) respectively, out of a possible 18. An independent-samples t-test found no significant difference between the two groups (t (88) = −1.33, *p* = 0.19, two-tailed) and the new teaching technique was considered non-inferior with a half of one SD margin.

### Retention score as outcome parameter

**Fig. 2:**
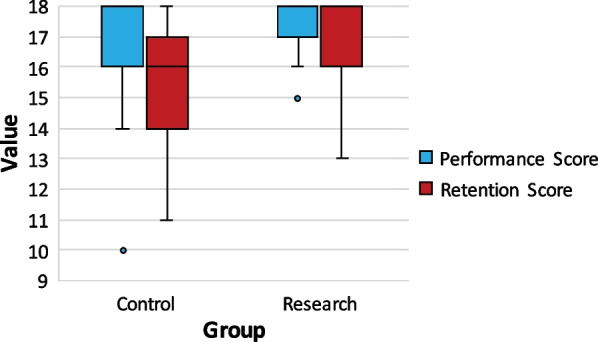
Performance and Retention scores for each group. Mean performance scores were not significantly different with 17.07 (SD = 1.58) and 17.43 (SD = 0.98) for the control and research group respectively. The independent-samples t-test showed significantly better (*p* < 0.001) mean retention scores for the research group 17.02 (SD = 1.33) compared to the control group 15.66 (SD = 1.86). *Note:* Horizontal bars show the median, upper and lower end of the box representing 1st and 3rd quartile. Narrow lines above and below the box represent the spread of values. Dots are outliers

The retention assessment was done two days after the teaching session, with mean scores for the control group at 15.66 (SD = 1.86) and 17.02 (SD = 1.33) for the research group. The difference in mean retention scores was statistically significant in favour of the research group (*p* < 0.001, 95% CI: −2.0 to −0.69, d = 0.85) (Fig. 2).

### Satisfaction as outcome parameter

#### Candidate satisfaction

The general satisfaction with the teaching session was measured on a scale of 1 (very satisfied) to 5 (very dissatisfied). Both groups reported 98% 'satisfied' or 'very satisfied', with a higher proportion of 'very satisfied' in the control group (77% vs. 59%). The Mann–Whitney U Test found no significant difference in overall satisfaction between the control group (Median (*Md*) = 1, n = 44) and the research group (*Md* = 1, n = 46), *U* = 1190, *z* = 1.76, *p* = 0.08.

Participants rated four aspects of the teaching session: explanation of indications for a pelvic binder, explanation of skill steps, instructor feedback, and practice time. Both groups reported over 90% 'satisfied' or 'very satisfied' for all items with no statistically significant differences between the groups for any of these items.

Eighty-six out of 90 candidates (96%) agreed that the performance assessment with the standardised checklist was objective.

#### Instructor satisfaction

24 instructors participated in the study, with 12 teaching the control group and 12 the research group (four instructors' data were excluded due protocol violation).

**Fig. 3:**
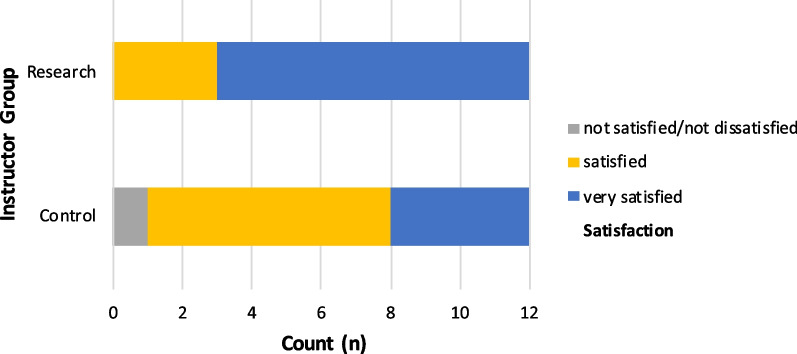
Instructor satisfaction with teaching session, by group. Satisfaction scores of instructors for the teaching technique they used was high for both groups, with no scores for unsatisfied or very unsatisfied. The research group reported a statistically significant higher proportion of very satisfied scores

Instructor satisfaction was measured on a 5-point Likert scale. Overall, 96% of instructors were satisfied or very satisfied (Fig. 3). Although both groups reported high levels of satisfaction, the research group had a higher proportion of 'very satisfied' instructors (75% versus 33%) and analysis with the Mann–Whitney U test revealed statistically significant higher satisfaction levels for the research group (*Md* = 1, *n* = 12) compared to the control group (*Md* = 2, *n* = 12), *U* = 40,500, *z* = -2.07, *p* = 0.038, *r* = 0.42.

All research group instructors rated time for feedback and practice as sufficient, while two instructors of the control group scored this time as insufficient. All instructors agreed that the performance assessment was objective.

Of the 12 instructors in the research group, 10 preferred the blended approach over the traditional teaching technique and 2 described it as equal.

#### Teaching time as outcome parameter

**Fig. 4:**
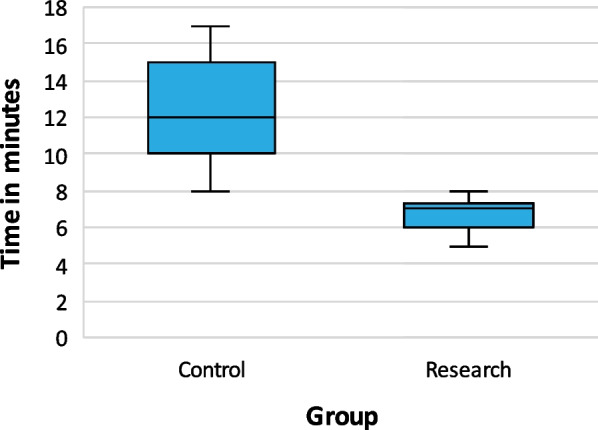
Mean teaching time per session, by group. Mean time of the traditional teaching session was 12.61min (SD = 2.80) and of research session 6.76min (SD = 0.99), showing a significant mean difference of 5.85 min (95% CI: 4.96 to 6.75, *p* < 0.001,* t* (13.11) between the two groups. Teaching time variability was smaller in the research group (5–8 min) compared to the control group (8–17 min). *Note*: Horizontal bars show the median, upper and lower end of the box representing 1st and 3rd quartile. Narrow lines above and below the box represent the spread of values

The study included 24 teaching sessions, 12 using traditional teaching and 12 using a blended approach. The traditional teaching sessions took a mean time of 12.61min (SD = 2.80), ranging from 8 to 17 min. The blended approach sessions took a mean time of 6.76min (SD = 0.99), ranging from 5 to 8 min (Fig. 4). Welch's t-test showed a significant mean difference of 5.85 min (95% CI: 4.96 to 6.75, *p* < 0.001,* t* (13.11) between the two groups, with a large effect size (*d* = *2.8*). The results indicate that the blended approach significantly reduced the teaching time compared to the traditional method.

## Discussion

The study confirmed that the blended teaching approach was non-inferior to the traditional method in skill performance, skill retention and satisfaction, whilst reducing face-to-face teaching time.

### Performance and retention scores

The results align with prior research showing no difference in performance scores when parts of the 4-step teaching technique are replaced by standardised videos [6, 14, 16, 17]. It innovatively uses a blended learning approach called the flipped classroom, where the first three steps of the 4-step technique are taught virtually [8]. Three recent meta-analyses [18–20] and a systematic review [21] reported better skill performance scores with the flipped classroom compared to traditional teaching.

In this study, replacing teaching steps with best-practice videos and interactive exercises, led to equivalent performance and an even better retention in the research group, although the mean difference in retention score of 1.36 out of 18 should be considered small. Interestingly, the skill demonstration fostered observational learning in the control group but seemed to stimulate knowledge recall in the research group [9, 22]. Repeated retrieval may enhance long-term retention [23], though evidence about the effect of blended learning on long-term retention is limited [24–26].

### Candidate satisfaction with the teaching session

Both teaching sessions were well received by the candidates, as 98% of each group reported high satisfaction levels. It should be noted that the control group reported a higher proportion of candidates (77%) to be 'very satisfied' compared to the research group (59%). In contrast to the present study results, students increasingly prefer to have control over their own learning so they can learn at their own pace and convenience [26, 27] and many already use open access web resources to complement their learning [26]. The more digital-proficient new generations increasingly expect digital technology to be incorporated in their education [28, 29] and recent literature reports an increasing preference for blended learning over sole face-to-face learning [26, 27, 30]. However, face-to-face teaching is still popular with students and teachers, mainly because of its familiarity and the absence of pre-course preparation [25, 31]. The absence of pre-course preparation could explain the small difference in satisfaction levels as many candidates attend an ETC in their own free time and do not appreciate additional preparation time.

### Instructor satisfaction with the blended technique

Instructors teaching the blended approach reported higher satisfaction levels, with 84% preferring this over the traditional 4-step technique. This mirrors the positive attitudes towards blended learning seen in other studies [21, 32]. To effectively implement this method, faculty must be informed about the educational foundation of the flipped classroom and the associated teaching transition from information provider to facilitator, a challenging shift that may require supportive training programs [33, 34]. Another challenge lies in creating standardised teaching content that assures quality while being adaptable to local needs [35]. Development of such materials will come with a considerable investment of time and funds [31, 36].

The current study noted lower candidate satisfaction in the research group, possibly due to instructor inexperience with the new approach. Some instructors struggled with the facilitator role or the standardised teaching content.

### Duration of the teaching session

Contrary to reports of worsened performance with reduced face-to-face time [31, 37], this study found that an, on average, 6-min shorter teaching session in the research group did not have negative effects on performance scores. This aligns with results reported by Baepler et al. [38] and implies a potential 36-min saving across six ETC skill teaching sessions. The reduced variability in teaching time measured for the research group (3 min) compared to the control group (9 min) favours more predictable course time management.

### Limitations

Although a standardised checklist was used which closely resembled the long-used ETC assessment criteria for a pelvic binder application and found consensus of an expert ETC panel, it was not officially validated, nor evaluated for its reliability. Face validity of the checklist appeared high, as 96% of the candidates and all the instructors rated the assessment with the checklist as objective.

The study results may not be generalisable to other technical skills, as effectiveness could be linked to skill complexity and specificity, and further evaluation is needed to confirm this.

Finally, the results may not be applicable to the entire ETC population, as the study was only conducted in two countries, Belgium and England, which could limit the universality of the findings.

## Conclusions

This multicentre, randomised controlled study evaluated the role of blended learning for teaching the application of a pelvic binder in the ETC, using a blended modification of the 4-step technique [3]. The results indicate that the blended approach could be a more standardised and time-efficient approach, matching technology-enhanced learning trends [26, 39, 40].

Future research should focus on the role of blended learning for skills with different complexity as the effectiveness of the 4-step technique and the blended modification, could be related to skill complexity [7, 41]. Further research is needed to evaluate long-term skill retention [4, 26] as well as the most effective online formats [21, 27] and the value of the 4-step instructional design for the blended approach.

As the blended approach requires the instructors to change from information providers to facilitators [34], a future qualitative study could tailor the new approach towards instructors' expectations and needs.

The findings of this study contribute to the growing body of evidence that demonstrates the effectiveness of the flipped classroom approach in transferring elements of skill learning to the virtual space [18–21]. With the increased use of even more advanced technologies like virtual reality and artificial intelligence [42, 43] there is the potential to further reduce the need for face-to-face teaching without compromising the quality of technical skill teaching. Ultimately, these new developments could help to increase resilience of teaching and learning, against evolving challenges.

### Supplementary Information

**Additional file 1.** Standardised checklist. This file shows the checklist which was used for the performance and retention assessment.

**Additional file 2.** Satisfaction Questionnaire for Candidates. This file shows the questionnaire which was used to measure the satisfaction of the candidates with the received teaching for pelvic binder application.

**Additional file 3.** Satisfaction Questionnaire for Instructors. This file shows the questionnaire which was used to measure the satisfaction of the instructors with the technique used for teaching pelvic binder application.

## Data Availability

All data and additional materials can be provided by the corresponding author upon reasonable request.

## Abbreviations

ETC: European trauma course
ILCOR: International liaison committee
ETCO: European trauma course organisation
