# Supplementary material for: From tradition to innovation: a comparison of the traditional 4-step approach versus a blended learning modification for technical skills teaching
Source: Scand J Trauma Resusc Emerg Med. 2023 Nov 14;31:80. doi: 10.1186/s13049-023-01127-4 (PMC10644658; doi:10.1186/s13049-023-01127-4)
Supplement: Supplementary file 1 — Additional file 1. Standardised checklist. This file shows the checklist which was used for the performance and retention assessment. [file 13049_2023_1127_MOESM1_ESM.pdf]

Standardised Checklist

Standardised Checklist for the examination of a PCCD application			
**Steps to be completed**	**Score**<br>(0=not done, 1=incorrectly done, 2=correctly done)		
	**0**	**1**	**2**
1. Considers adequate pain relief			
2. Feels for peripheral pulses			
3. Localises greater trochanters			
4. Positions the PCCD correctly at the greater trochanter level			
5. Closes the binder correctly (click noise)			
6. Secures the binder with the Velcro strap			
7. Re-assessment of peripheral pulses			
8. Binds the feet together to decrease external rotation			
9. Documents the time of application			
**Total score (out of 18):**			
