# Supplementary material for: From tradition to innovation: a comparison of the traditional 4-step approach versus a blended learning modification for technical skills teaching
Source: Scand J Trauma Resusc Emerg Med. 2023 Nov 14;31:80. doi: 10.1186/s13049-023-01127-4 (PMC10644658; doi:10.1186/s13049-023-01127-4)
Supplement: Supplementary file 2 — Additional file 2. Satisfaction Questionnaire for Candidates. This file shows the questionnaire which was used to measure the satisfaction of the candidates with the received teaching for pelvic binder application. [file 13049_2023_1127_MOESM2_ESM.pdf]

Candidate Satisfaction Questionnaire

Pelvic Circumferential Compression Device (PCCD) Study<br>Questionnaire – Candidate Satisfaction<br>Please circle the preferred answer
Date:<br>Previous exposure to teaching of a pelvic binder application (excl. online module): Y / N<br>Time needed for online module: N/A 0-15 min. 15-30 min. > 30min.
**The teaching session (incl. online module) for the application of a pelvic binder provided:**
The indications for pelvic binder application<br>1=Completely agree 2=agree 3=not agree/not disagree 4=disagree 5=completely disagree
A clear explanation about the steps necessary to correctly apply a pelvic binder<br>1=Completely agree 2=agree 3=not agree/not disagree 4=disagree 5=completely disagree
Constructive feedback on my performance<br>1=Completely agree 2=agree 3=not agree/not disagree 4=disagree 5=completely disagree
Sufficient time for practicing the application of a pelvic binder<br>1=Completely agree 2=agree 3=not agree/not disagree 4=disagree 5=completely disagree
An objective assessment of my performance<br>1=Completely agree 2=agree 3=not agree/not disagree 4=disagree 5=completely disagree
My satisfaction with this teaching session<br>1=very satisfied 2=satisfied 3=not satisfied/not dissatisfied 4=dissatisfied 5=very dissatisfied
**Thank you for completing this Questionnaire!**
