# Supplementary material for: From tradition to innovation: a comparison of the traditional 4-step approach versus a blended learning modification for technical skills teaching
Source: Scand J Trauma Resusc Emerg Med. 2023 Nov 14;31:80. doi: 10.1186/s13049-023-01127-4 (PMC10644658; doi:10.1186/s13049-023-01127-4)
Supplement: Supplementary file 3 — Additional file 3. Satisfaction Questionnaire for Instructors. This file shows the questionnaire which was used to measure the satisfaction of the instructors with the technique used for teaching pelvic binder application. [file 13049_2023_1127_MOESM3_ESM.pdf]

Instructor Satisfaction Questionnaire

**Pelvic Circumferential Compression Device (PCCD) Study**<br>**Questionnaire – Instructor Satisfaction**<br>**Please circle the preferred answer**
Date:
**The teaching session for the application of a pelvic binder provided:**
Sufficient time for corrective feedback<br><br>1=Completely agree 2=agree 3=not agree/not disagree 4=disagree 5=completely disagree
Sufficient time for candidates to practise the application of a pelvic binder<br><br>1=Completely agree 2=agree 3=not agree/not disagree 4=disagree 5=completely disagree
An objective assessment with the standardised checklist<br><br>1=Completely agree 2=agree 3=not agree/not disagree 4=disagree 5=completely disagree
My satisfaction with this teaching session<br><br>1=very satisfied 2=satisfied 3=not satisfied/not dissatisfied 4=dissatisfied 5=very dissatisfied
Compared to the traditional skill teaching, I find the blended approach:<br><br>Better / Equal / Worse
**Thank you for completing this Questionnaire!**
